# Supplementary material for: Nanofluidic voidless electrode for electrochemical capacitance enhancement in gel electrolyte
Source: Nat Commun. 2021 Sep 17;12:5515. doi: 10.1038/s41467-021-25817-8 (PMC8448854; doi:10.1038/s41467-021-25817-8)
Supplement: Supplementary file 1 — Supplementary Information [file 41467_2021_25817_MOESM1_ESM.pdf]

Supplementary Information

Nanofluidic voidless electrode for electrochemical capacitance enhancement in gel electrolyte

Kefeng Xiao[1], Taimin Yang[2], Jiaxing Liang[1], Aditya Rawal[3], Huabo Liu[1], Ruopian Fang[1], Rose Amal[1], Hongyi Xu[2]*, Da-Wei Wang[1]*

[1] School of Chemical Engineering, The University of New South Wales, Sydney, NSW 2052, Australia.

[2] Department of Materials and Environmental Chemistry, Stockholm University, Stockholm 10691, Sweden.

[3] Nuclear Magnetic Resonance Facility, Mark Wainwright Analytical Center, The University of New South Wales, Sydney, NSW 2052, Australia.

*Correspondence to: da-wei.wang@unsw.edu.au, hongyi.xu@mmk.su.se

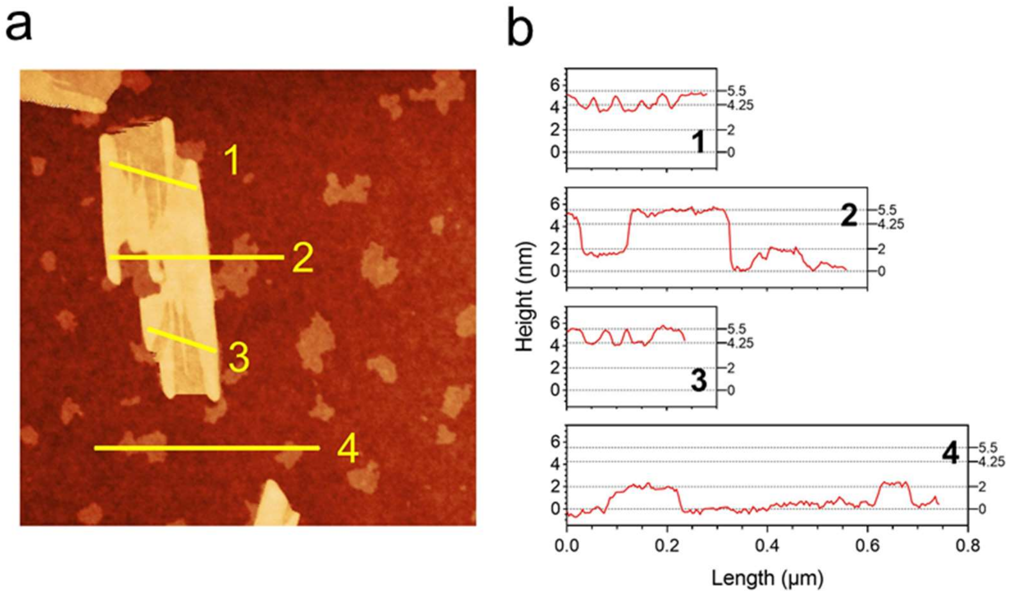

Supplementary Fig. 1 a AFM image of exfoliated TALP nanosheets. **b** Height profile of the targeted areas under tapping mode.

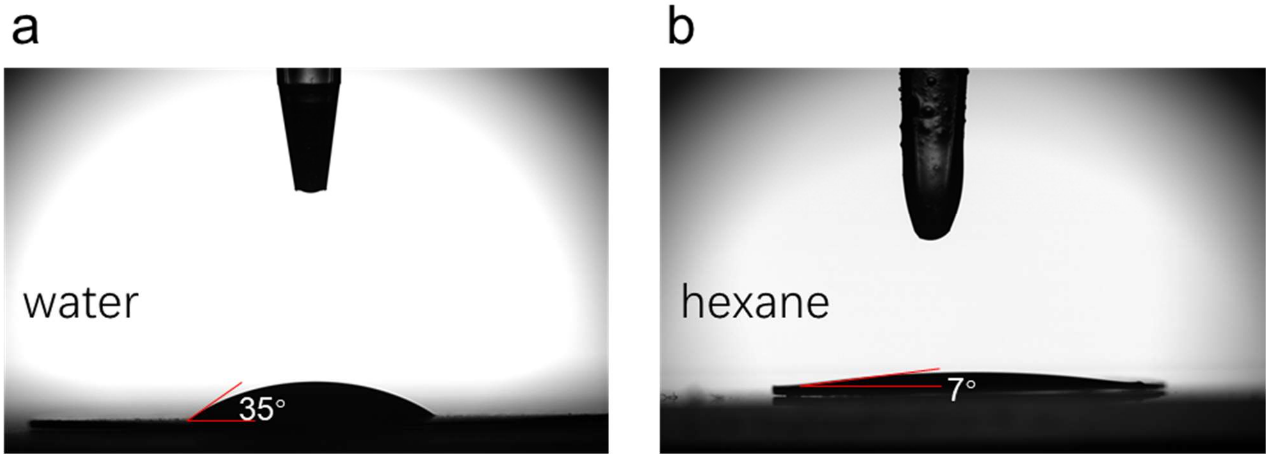

Supplementary Fig. 2 Contact angles of **(a)** water and **(b)** hexane on TALP pellet surface**.**

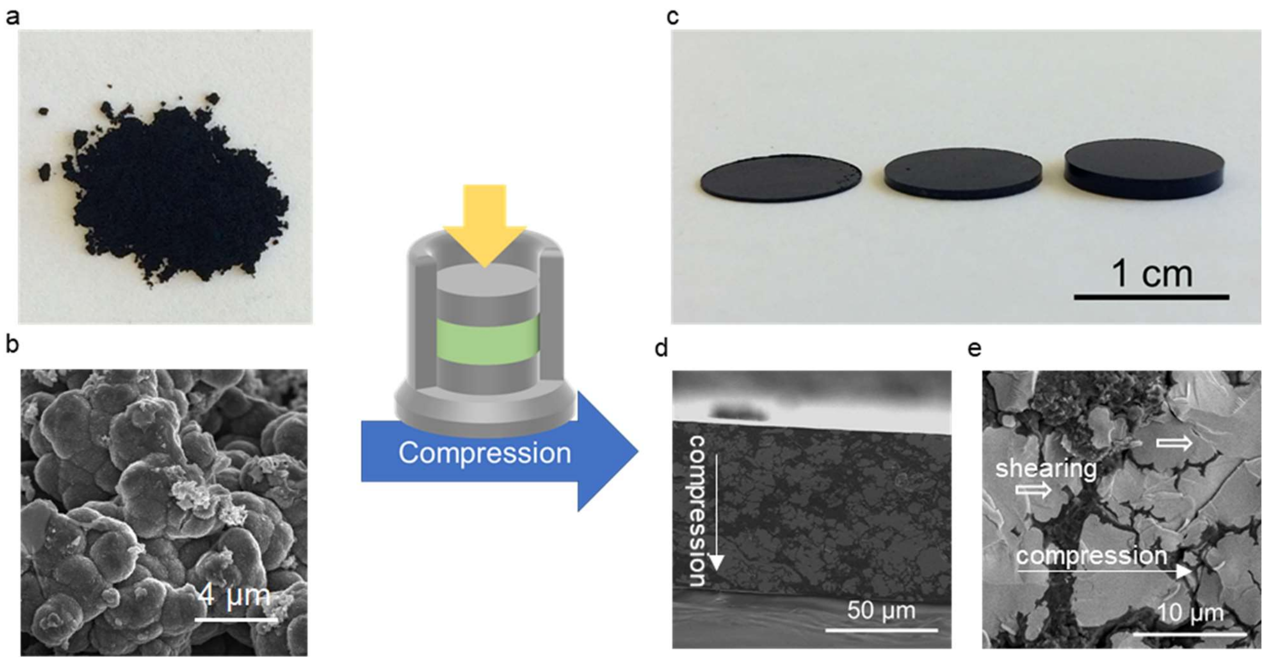

Supplementary Fig. 3 a The digital photo of the TALP powders. **b** SEM image of aggregated TALP particles. **c** The digital photo of the compacted TALP pellets with different thicknesses and mass loadings. **d** The SEM image of the cross-sectional surface of the TALP pellet. **e** The zoom-in SEM image of the cross-sectional surface of the TALP pellet showing the sliding of the sheared nanosheets.

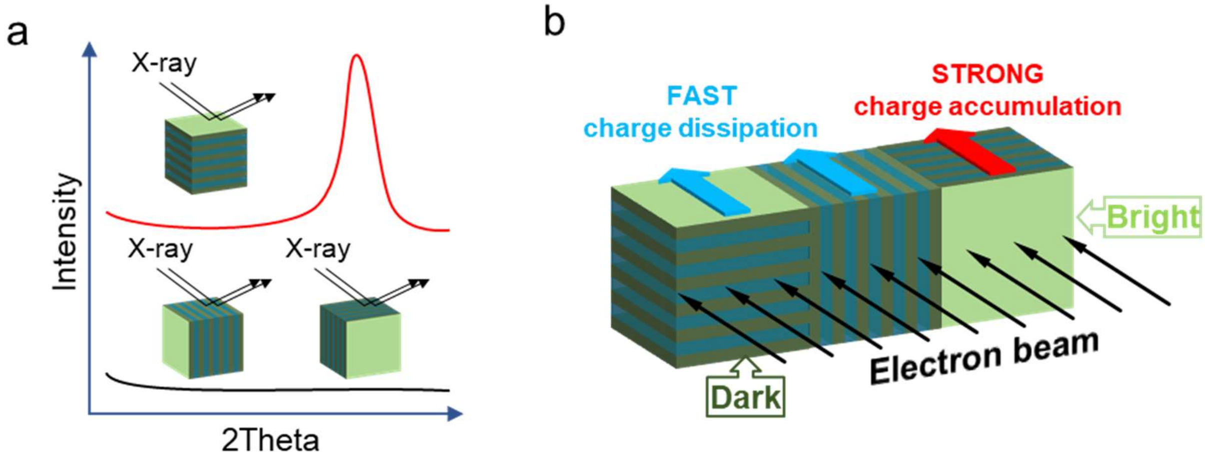

Supplementary Fig. 4 a Schematic illustration of the X-ray incident direction and the diffraction intensity as a result of the nanofluidic channel orientation in compacted TALP pellet. **b** Hypothetical illustration of the relationship between the preferential alignment and the surface contrast under electron beam irradiation. Bright area is attributed to the basal planes of the TALP due to their surface charging and smoothness. Dark area is related to the edge planes of the TALP because of the fast charge conduction.

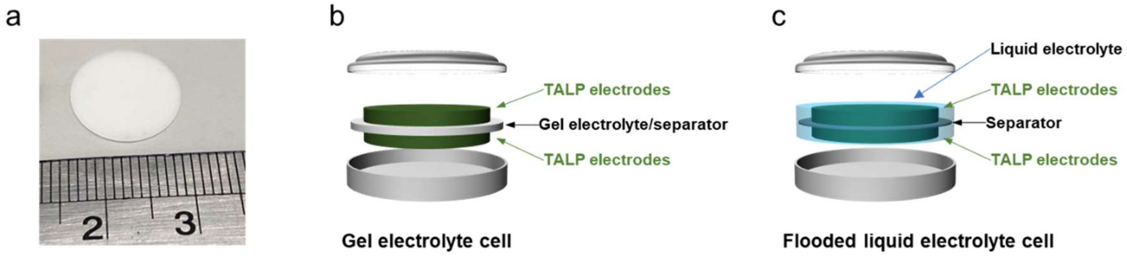

Supplementary Fig. 5 a A photo of the dry, leak-free solid gel electrolyte membrane. **b** An illustration of the two-electrode solid-state EC cell. The solid gel electrolyte was directly sandwiched by two TALP pellets. The pellets were neither soaked in liquid electrolyte nor prefilled with gel before cell assembly. Note that the interphase between the nanofluidic TALP electrodes and the gel electrolyte membrane is most likely in hydrated states due to the hydrophilicity of both components. The interfacial affinity readily allows the ions hop forward and backward. After crossing the interphase, the ions can rapidly move around in the gel-free TALP electrodes through the percolating 2D nanofluidic channels. Further enhancement of the solid-state ion kinetics is achievable dependent on the control and improvement of the texture of nanofluidic channels and the gel/TALP interphase chemistry. **c** An illustration of the two-electrode liquid-state EC cell with flooded liquid electrolyte.

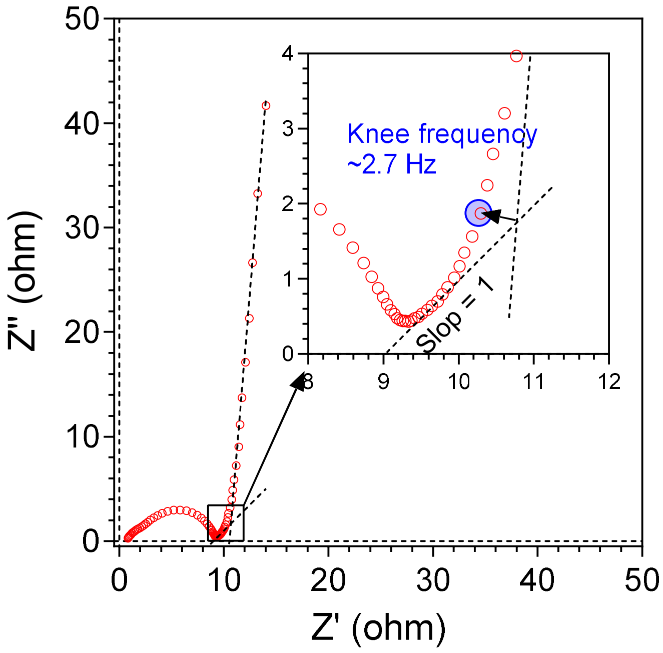

Supplementary Fig. 6 The knee frequency of 2.7 Hz is located through the cross point of tangent line of the diffusion part of the Nyquist curve with slop of 1 and the tangent line of the tail part of the Nyquist curve.

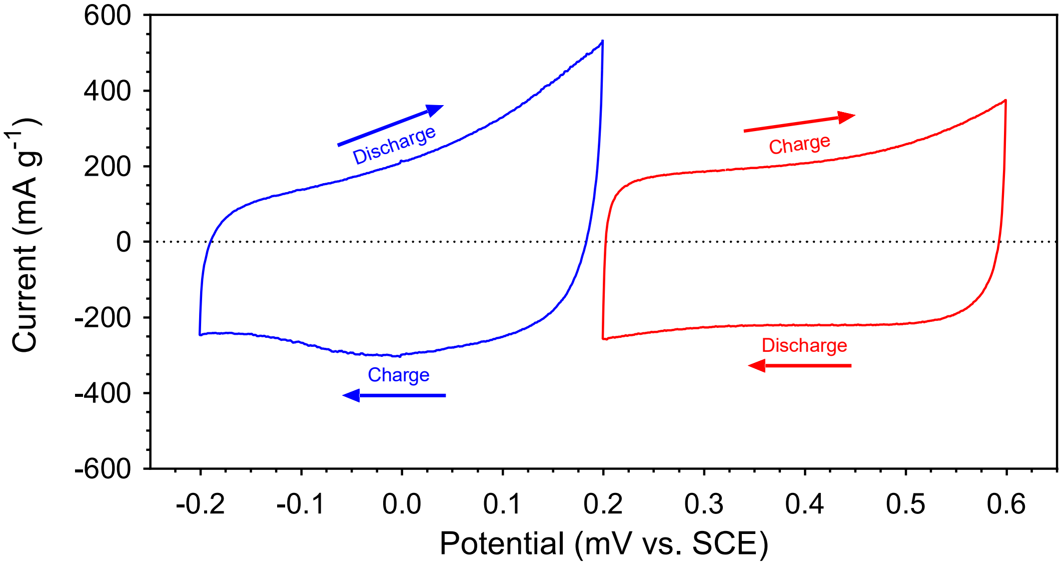

Supplementary Fig. 7 CV curves (at 2mV/s) of positive and negative electrodes using a three-electrode system in 1M aqueous solution of Na_2SO_4 with SCE as reference electrode.

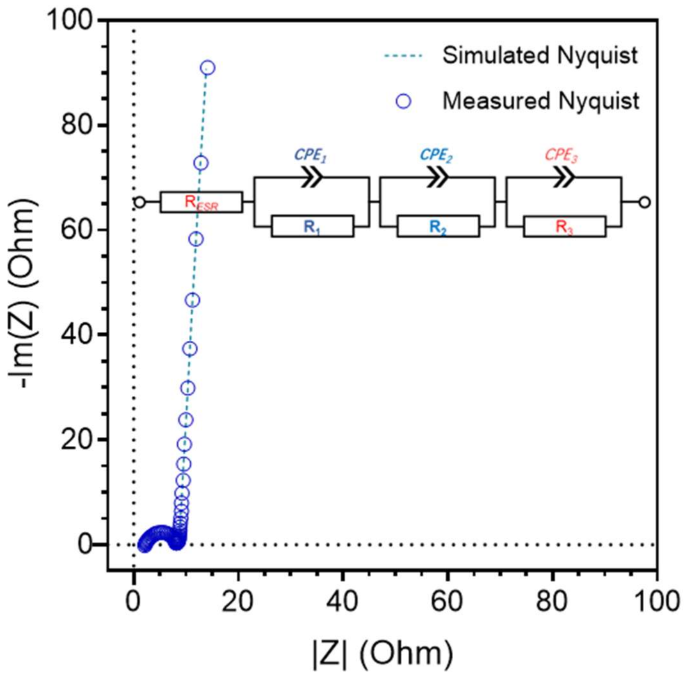

Supplementary Fig. 8 Equivalent circuit model for TALP pellet electrodes. The cell resistances were divided to four parts: the equivalent serial resistance (ESR), the interfacial ion resistance (R_1), the intra-particle ion resistance (R_2), and the inter-particle ion resistance (R_3). Rate dependence of

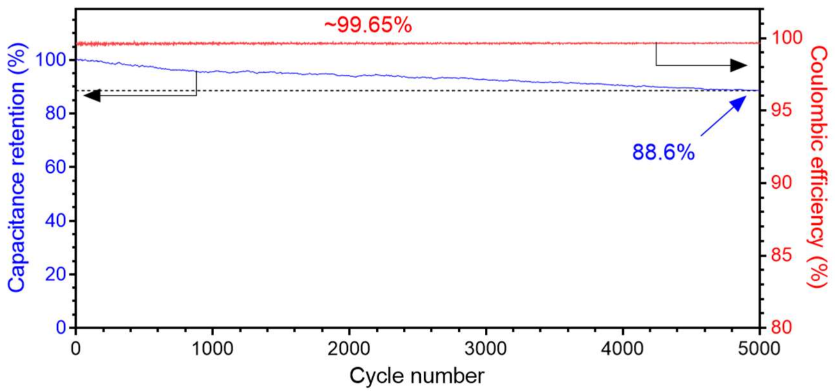

Supplementary Fig. 9 Cyclic performance of TALP electrode in gel electrolyte (20 mA cm^{-2}). All tests for gel electrolyte were conducted in a two-electrode solid state EC cell as illustrated in Fig. 5b.

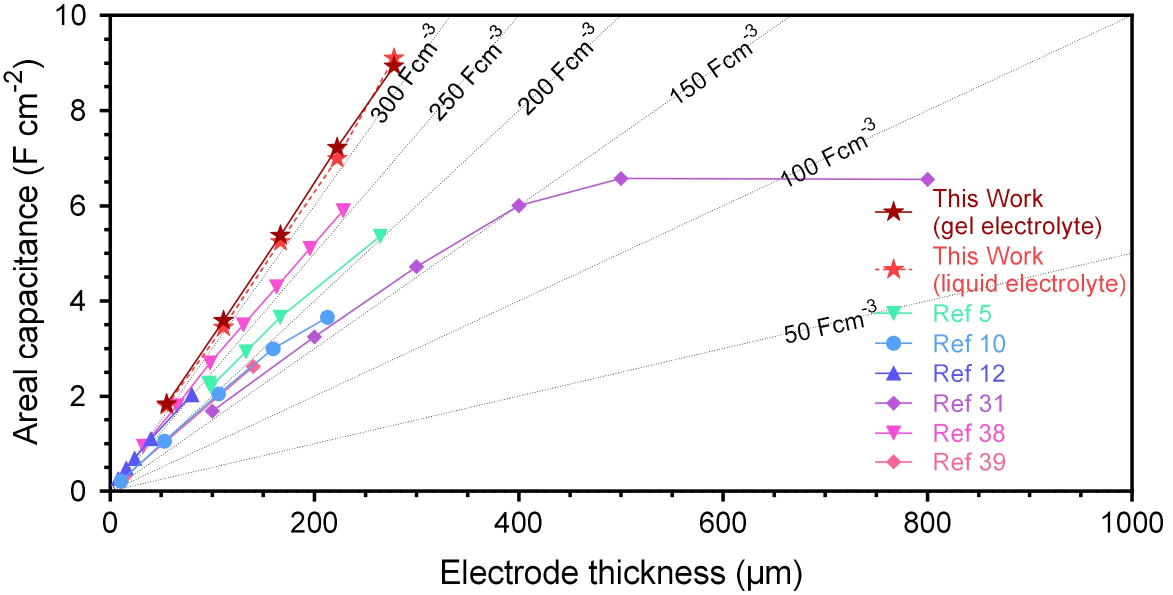

Supplementary Fig. 10 The areal capacitance variation of EC electrode to the thickness of electrode [5, 10, 31, 38, 39] (references in main text).

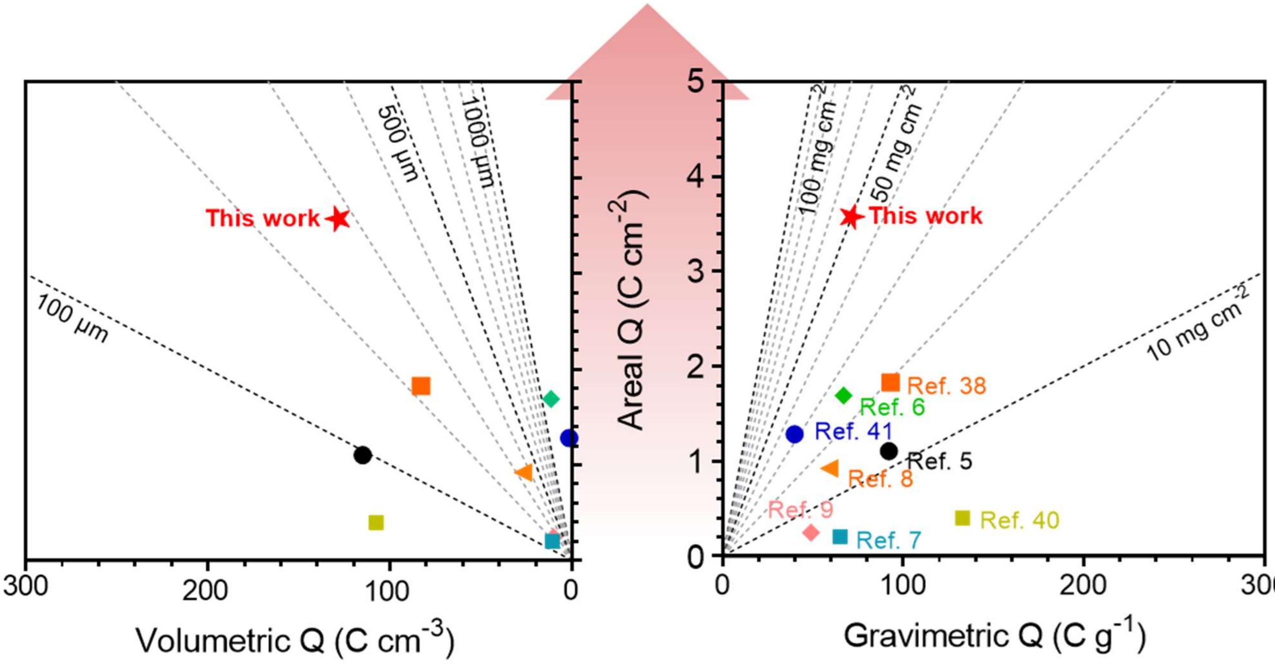

Supplementary Fig. 11 A holistic comparison of the quantity of Charge (Q) of the nanofluidic electrode with gel electrolyte (this work) and gel-infilled porous electrodes for areal, volumetric and gravimetric metrics[5-9,38,40,41] (references in main text). The Charge is calculated from the capacitance in Fig. 4a and the electrode voltage change reported in the corresponding references.

Supplementary Table 1 Resource data for Figure 4a

	Capacitance			Voltage (V)	Electrode material	Electrolyte
	Areal ($F\ cm^{-2}$)	Gravimetric ($F\ g^{-1}$)	Volumetric ($F\ cm^{-3}$)			
This Work	8.94	178.8	321.8	0.8	TALP	PVA/PEG/Na_2SO_4
Ref. 5	2.208	184	230	1	Graphene	PVA/H_2SO_4
Ref. 6	3.38	134	22.32	1	active carbon	PVA/KOH
Ref. 7	0.402	130	21.7	1	Graphene	PVA/H_2SO_4
Ref. 8	1.84	117.95	54.12	1	microporous carbon	PVA/H_2SO_4
Ref. 9	0.816	162.8	34	0.6	MXene/Mof	PVA/H_2SO_4
Ref. 38	3.65	185.9	166.1	1	Graphene	PVA/KOH
Ref. 40	0.995	332	268.9	0.8	PAni/CNT	PVA/H_2SO_4
Ref. 41	2.56	79.5	3.2	1	GO/rGO/CNT	PVA/H_2SO_4

Supplementary Table 2 Resource data and translated total gravimetric capacitance for typical porous electrodes.

	Density (g cm^{-3})	Porosity (%)	Electrode mass loading (mg cm^{-2})	Electrolyte uptake (mg cm^{-2})	Performance factor (%)	Gravimetric capacitance (F g^{-1})	Total gravimetric capacitance (F g^{-1})
Ref.5 (Graphene)	1.25	43.18%	12	6.218	65.87%	184	121.2
Ref.7 (Graphene)	0.167	92.41%	3.1	25.731	10.75%	130	13.9
Ref.8 (Microporous carbon)	0.459	79.14%	15.6	40.344	27.89%	118	32.9
Ref.10 (Graphene)	0.94	57.27%	1	0.914	52.25%	166	86.7

Supplementary Table 3| Calculation methods for translated total gravimetric capacitance in table S1.

Porosity calculation	
$$\mathbf{P = 1 - \frac{\rho}{\rho_0}}$$	P = Porosity of electrode<br>ρ = Bulk density of electrode<br>ρ_0 = Real density of material (2.2 for carbon)
Electrolyte uptake	
$$m_E = \frac{m}{\rho} \times P \times \rho_E$$	m_E = Mass of electrolyte uptake<br>m = Mass loading of electrode<br>ρ_E = Density of electrolyte (1.5 g cm^{-3} based on PVA/H_2SO_4)
Performance factor	
$$f_p = \frac{C_t}{C} = \frac{m}{m + m_E}$$	C_t = Total gravimetric capacitance (electrode and electrolyte uptake)<br>C = Gravimetric capacitance of electrode<br>f_p = Performance factor

Supplementary Table 4 The mass loading, thickness and areal capacitance of TALP electrode

	Mass loading ($mg\ cm^{-2}$)	Thickness (μm)	Areal capacitance ($F\ cm^{-2}$)		
			$1\ mA\ cm^{-2}$	$10\ mA\ cm^{-2}$	$30\ mA\ cm^{-2}$
Liquid electrolyte	10	56	1.80	1.28	0.89
	20	111	3.45	2.35	1.61
	30	167	5.25	3.57	2.39
	40	222	6.99	4.50	2.89
	50	278	9.10	6.28	3.92
Gel electrolyte	10	56	1.84	1.27	0.72
	20	111	3.59	2.36	1.40
	30	167	5.38	3.29	1.88
	40	222	7.23	4.25	2.37
	50	278	8.94	5.30	3.06

Supplementary Note

An example calculation of instantaneous and average capacitance

The practical calculation given below is based on real experimental data, where the *dt* and *dV* experimentally determined by t_{n+1}-t_n and V_{n+1}-V_n, respectively. The C_{ins} and C_{ave} can be calculated by the following equations:

$$C_{ins} = \frac{I \times dt}{dV} = \frac{I \times (t_{n+1} - t_n)}{V_{n+1} - V_n} \quad (1)$$

$$C_{ave} = \frac{1}{V} \int C_{ins} dV = \frac{1}{V} \sum (C_{ins} \times (V_{n+1} - V_n)) \quad (2)$$

Where, C_{ins} represents the instantaneous capacitance of the segmental voltage window (*dV*), *I* represents the current density applied in GCD test, *dt* represents the segmental period of discharge, *dV* represents the corresponding segmental voltage window, t_n represents the time data collected (n=1, 2, 3 …), and the V_n represents the voltage data collected (n=1, 2, 3 …).

Herein, we use the discharge curve under current of 1 mA cm^{-2} of a symmetric TALP cell with gel electrolyte and single electrode mass loading of 10 mg cm^{-1} to calculate the C_{ins} and C_{ave}.

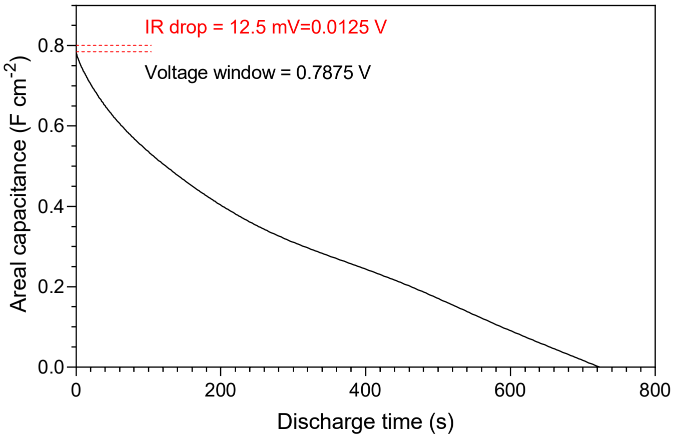

The plot of *C* vs. *V* is shown as the following figure:

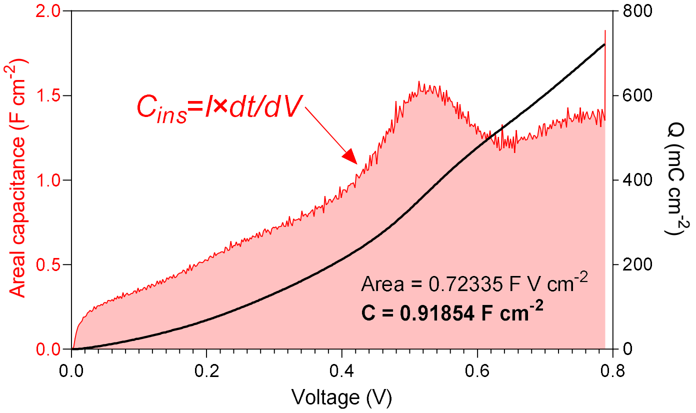

The average areal capacitance of the symmetric TALP cell in the voltage window of 0.7875 V is calculated as 0.91854 F cm^{-2} through equations (1) and (2). This value deviates negligibly from the value (0.91850 F cm^{-2}) calculated by $C=I\times t/V$, where t is the total discharge time and V is the whole discharge voltage window.
